# Development of a City-wide Rapid Antiretroviral Therapy Initiation Toolkit for People Newly Diagnosed With HIV in the Southern United States

**DOI:** 10.1093/ofid/ofae660

**Published:** 2024-11-20

**Authors:** A C Pettit, A A Ahonkhai, L Pierce, P F Rebeiro, C M Valdebenito, J Woods, L Gregory, C Walton, R Nash, N A Summers, A Van Wylen, D Thompson, M Hayes-Winton, A Eke, L C Pichon, C M Audet

**Affiliations:** Department of Medicine, Vanderbilt University Medical Center, Nashville, Tennessee, USA; Department of Medicine, Massachusetts General Hospital, Boston, Massachusetts, USA; Department of Medicine, Massachusetts General Hospital, Boston, Massachusetts, USA; Department of Medicine, Vanderbilt University Medical Center, Nashville, Tennessee, USA; University of Memphis School of Public Health, Division of Social and Behavioral Sciences, Memphis, Tennessee, USA; Angelic Branding Consulting, Nashville, Tennessee, USA; Tennessee Department of Health, Nashville, Tennessee, USA; Tennessee Department of Health, Nashville, Tennessee, USA; Tennessee Department of Health, Nashville, Tennessee, USA; Department of Medicine, University of Tennessee Health Science Center, Memphis, Tennessee, USA; Christ Community Health Services, Memphis, Tennessee, USA; Christ Community Health Services, Memphis, Tennessee, USA; Shelby County Health Department, Memphis, Tennessee, USA; Division of HIV Prevention, Centers for Disease Control and Prevention, Atlanta, Georgia, USA; University of Memphis School of Public Health, Division of Social and Behavioral Sciences, Memphis, Tennessee, USA; Department of Medicine, Vanderbilt University Medical Center, Nashville, Tennessee, USA; Department of Health Policy, Vanderbilt University Medical Center, Nashville, Tennessee, USA

## Abstract

**Background:**

Rapid antiretroviral therapy (ART) initiation, in which individuals with HIV start treatment within days of diagnosis, is a key component of the United States (US) Ending the HIV Epidemic initiative. The Memphis Metropolitan Statistical Area ranks second in the US for HIV incidence, yet only ∼60% of individuals link to treatment within 1 month of diagnosis. This study aimed to identify barriers and strategies for implementing rapid ART initiation in Memphis.

**Methods:**

From August to December 2022, we conducted process mapping guided by the Consolidated Framework for Implementation Research to outline the steps from 3 HIV testing sites to ART prescription at 3 Ryan White-funded clinics in Memphis, Tennessee. We used modified conjoint analyses to prioritize barriers and identify strategies for improving rapid ART implementation, focusing on the importance and feasibility of changes.

**Findings:**

Prioritized barriers included intersectional stigma and a lack of access to centralized information about the rapid ART program, branding and logo development, inter- and intra-organizational networking and communication, testing and treatment resources (HIV testing kits and ART starter packs), rapid ART knowledge, and organizational champions. Strategies to address these barriers were compiled into a local rapid ART toolkit.

**Conclusions:**

We identified modifiable systemic barriers to rapid ART initiation in Memphis, a community disproportionately affected by HIV. The strategies developed to address these barriers informed the creation of a locally relevant rapid ART toolkit for future evaluation. These methods could be applied in other high-burden areas seeking to develop local rapid ART models.

## Teaser

Implementation science methods, including process mapping and conjoint analyses, identified key modifiable barriers and strategies for rapid ART initiation in Memphis, Tennessee—a community disproportionately affected by HIV. These methods can inform relevant rapid ART initiation programs in other settings.

The United States Ending the HIV Epidemic (EHE) Initiative aims to decrease incident HIV by 75% within 5 years and 90% within 10 years [1]. Rapid antiretroviral therapy (ART) initiation, or the initiation of ART as soon as possible following HIV diagnosis, has been shown to prevent HIV transmission among sexual partners [2]. Therefore, the US Department of Health and Human Services [3], World Health Organization [4], and International Antiviral Society-USA [5] all endorse rapid ART initiation as an important tool to end the HIV epidemic.

Memphis, Tennessee, is located in the US South, a region of the US disproportionately impacted by the HIV epidemic, and currently ranks second among US Metropolitan Statistical Areas for HIV incidence (26.4 per 100 000 persons) [6]. Further, Memphis is the largest majority-Black city in the United States, with 52% of its population belonging to this racial group [7]. The city's demographic composition contributes to its high ranking in HIV incidence, reflecting structural factors, including racial discrimination, driving HIV racial inequities nationally. Linkage to HIV care in Memphis remains poor, with ∼60% of people newly diagnosed with HIV linking to care within 1 month of HIV diagnosis—well below the 90% US EHE goal and other high-incidence Southern Metropolitan Statistical Areas (MSAs) including Miami (82%), Atlanta (83%), New Orleans (90%), Houston (75%), and Dallas (77%) [6]. Implementing rapid ART has the potential to improve HIV linkage and care outcomes in Memphis.

Although several rapid ART toolkits exist [8–10], tailoring is essential to ensure cultural relevance, address local needs and resource availability, and foster community buy-in and trust. Implementation science (IS) is a multidisciplinary specialty that seeks to understand the lack of uptake of evidence-based interventions in routine practice and to identify contextually appropriate strategies to close evidence-practice gaps [11]. We assembled a multidisciplinary team consisting of academic, implementation, and community partners to employ IS methods to identify barriers and develop strategies to facilitate rapid ART initiation in Memphis, Tennessee. Our primary objective was to create a rapid ART toolkit tailored to Memphis, while also sharing our methods to assist other high-burden US jurisdictions in systematically developing or refining their own contextually appropriate rapid ART models.

## METHODS

### Study Setting

Utilizing public health surveillance data from the Tennessee Department of Health, we identified 3 implementing partners that detected more than half of all individuals newly diagnosed with HIV in Memphis: (1) a community-based organization, (2) a faith-based federally qualified health center, and (3) the municipal public health department. Similarly, we identified 3 implementing partners providing longitudinal HIV care to more than half of PWH in Memphis: 2 providing adult HIV care (the same federally qualified health center providing high-volume testing noted previously and an adult university-affiliated dedicated HIV clinic) and a pediatric infectious diseases specialty clinic that provides all pediatric HIV care in Memphis. The study team consisted of academic partners with expertise in HIV clinical care, IS, public health, biostatistics, epidemiology, and community-based participatory research, implementing partners from participating HIV testing and treatment site leadership and staff, and community partners from the Memphis Connect-2-Protect (C2P) Community HIV Coalition [12]. C2P is a longstanding community-based coalition consisting of members from local HIV service organizations, community- and faith-based organizations, local and state government, public schools, federally qualified health centers, and people with HIV (PWH). This project was reviewed and determined to be exempt from human subjects research by the Vanderbilt University Medical Center institutional review board.

### Rapid ART Barrier Identification

Process mapping was conducted at all 5 participating HIV implementing sites that were purposively selected, from August to December 2022. Process mapping is a systems engineering technique in which physical maps are drawn by healthcare workers of the paths their patients take during clinical care; process mapping helps identify rate-limiting steps and brings staff to consensus on the systems-level processes their patients navigate [13]. Academic partners conducted process mapping during a first round of staff meetings at implementing sites, using a Consolidated Framework for Implementation Science Research (CFIR) guide, to collect preimplementation data on the barriers and facilitators to rapid ART initiation in Memphis. Results from process mapping were summarized using the CFIR to describe barriers and facilitators to rapid ART initiation systematically. The CFIR allows researchers to select domains and constructs that are associated with the successful implementation of evidence-based interventions. The study team aimed to identify constructs (facilitators and barriers) within each of the 5 CFIR domains: Intervention Characteristics, Inner Setting (eg, organizational structure, politics, culture, networks), Outer Setting (societal contexts—economic, political, and social), Characteristics of Individuals, and Implementation Process (planning) [14]. The selection of constructs within the CFIR domains was guided by available literature, existing rapid ART toolkits, and the knowledge of current Memphis ART initiation processes among study team members, including academic, implementing, and community partners in the Memphis C2P Community HIV Coalition [12].

### Rapid ART Barrier Ranking

**Figure 1.**
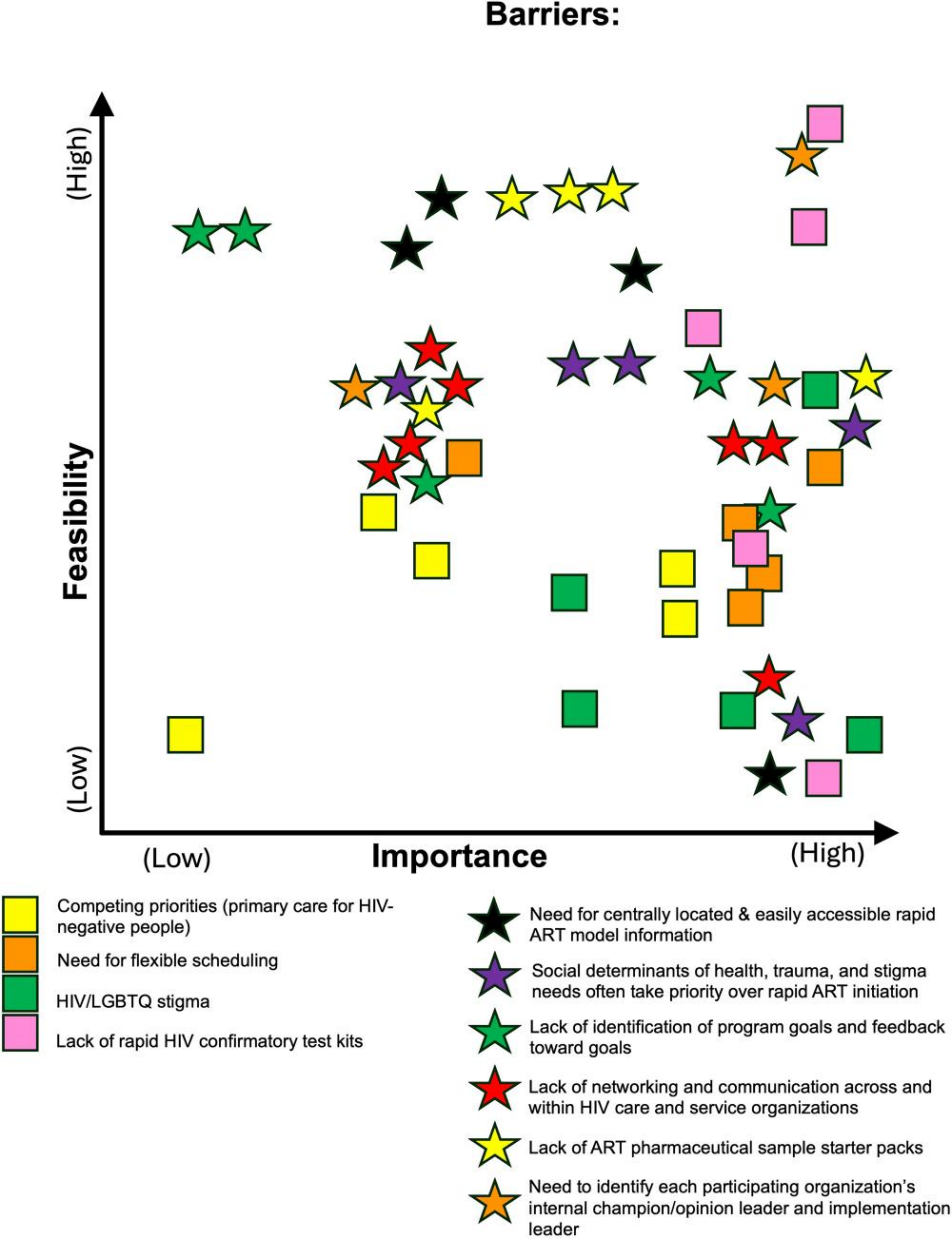
Modified conjoint analysis result example, Memphis/Shelby County, Tennessee 2023. Yellow square: Competing priorities (primary care for HIV-negative people); orange square: need for flexible scheduling; green square: HIV/LGBTQ stigma; pink square: lack of rapid HIV confirmatory test kits; black star: need for centrally located and easily accessible rapid ART model information; purple star: social determinants of health, trauma, and stigma needs often take priority over rapid ART initiation; red star: lack of networking and communication across and within HIV care and service organizations; green star: lack of identification of program goals and feedback toward goals; orange star: need to identify each participating organization's internal champion/opinion leader and implementation leader; yellow star: lack of ART pharmaceutical sample starter packs.

In a second round of staff meetings held in 2023, academic partners presented a list of 10 barriers identified most frequently during process mapping to the staff at each implementing site. Implementing site staff were asked to either confirm their agreement with the academic partners' list of barriers or revise it until they reached a consensus on a final list of the 10 most frequently encountered barriers. Using this final list, the academic partners then conducted a modified conjoint analysis, a quantitative method wherein implementing site staff assigned values to barriers to rank them based on their importance and feasibility of change [15]. Each barrier was assigned a colored Post-It tab or marker and implementing site staff were asked to place each barrier on a 4-quadrant grid that quantified importance on the horizontal axis and feasibility on the vertical axis (Figure 1). Barriers that were assigned both high feasibility and high importance rankings (upper right quadrant) were considered top priorities for the identification of implementation strategies to address those barriers. Barriers were not further ranked within each of the 4 quadrants.

### Rapid ART Implementation Strategy Selection

The study team utilized several approaches to identify potential rapid ART implementation strategies in 2023. First, academic partners gathered input from implementing site staff on potential strategies during both the process mapping and modified conjoint analysis activities. Strategies were prioritized for action based on how frequently they were identified, as well as their perceived feasibility and potential impact. Second, academic and implementing partners presented the process mapping and modified conjoint analysis results to the Memphis C2P Community HIV Coalition for feedback. Members of the Memphis C2P coalition served as the local Memphis EHE Planning Group. This plan, *End HIV 901* [12], outlined several potential rapid ART implementation strategies based on best practices from the published literature and existing toolkits to address the prioritized barriers identified.

## RESULTS

### Rapid ART Barrier and Facilitator Identification

Through process mapping activities during staff meetings at implementing sites, academic and implementing partners identified 1 to 5 barriers and/or facilitators per CFIR construct to balance comprehensiveness and feasibility. The Inner Setting was the CFIR Domain for which the most barriers and/or facilitators were identified. Similarly, the Implementation Climate construct, within the Inner Setting domain, was the CFIR construct with the most barriers and/or facilitators identified (Table 1).

**Table 1.** CFIR Domains, Constructs, and Rapid ART Facilitators and Barriers Identified During Process Mapping Activities Conducted With Implementing HIV Testing and Treatment Partners in Memphis/Shelby County, Tennessee, 2022

CFIR Domains and Constructs	Facilitator^a^	Barrier^a^
Intervention Characteristics		
Design Quality and Packaging	…	Need for centrally located and easily accessible rapid ART model information Need for local program branding
Cost	…	Balancing new programs and current internal program benchmarks (eg, client cycle time)
Outer Setting		
Patient Needs and Resources	Patients desire rapid ART initiation	Social determinants of health, trauma, and stigma needs often take priority over rapid ART initiation
Cosmopolitanism	…	Lack of networking and communication across HIV care and service organizations
Peer Pressure	Successful city-wide rapid ART programs (eg, San Francisco, Atlanta)	…
External Policies and Incentives	Local and national EHE plans and guidelines prioritize rapid ART	Lack of tangible incentives; no mechanism to require groups/clinics to facilitate rapid ART initiation.
Inner Setting		
Structural Characteristics	…	Stigma related to being a government agency (fear of government involvement in healthcare), dedicated HIV clinic (fear of being “outed”), faith-based institution (fear of being stigmatized for living with HIV/LGBTQ+ identity) Policies related to hiring and salary determination
Culture	…	Staff turnover/burnout and low morale, particularly among those employed by a municipal health department
Networks and Communication	…	Lack of networking and communication within HIV care and service organizations
Implementation Climate	…	Competing priorities (eg, primary care for HIV-negative people, COVID and Mpox epidemics) Lack of provision of HIV care at some testing sites (referrals for appointments at HIV treatment centers are seen as frustrating) Lack of identification of program goals and feedback toward goals
Implementation Readiness	…	Lack of resources (eg, flexible scheduling templates, rapid HIV confirmatory test kits, ART starter pack samples)
Characteristics of Individuals		
Knowledge and Beliefs	HIV and rapid ART initiation trainings are readily available	Available knowledge is not routinely accessed
Process		
Engaging	Memphis C2P Community HIV Coalition engagement	Need to identify each participating organization's internal champion/opinion leader and implementation leader
Reflecting and Evaluating	…	Lack of venue to share successes and challenges and refine rapid ART model

Abbreviations: ART, antiretroviral therapy; C2P, Connect-2-Protect; CFIR, Consolidated Framework for Implementation Science Research; EHE, Ending the HIV Epidemic Initiative; LGBTQ, lesbian, gay, bisexual, transgender, queer/questioning.

^a^Barriers and facilitators are listed in no particular rank order.

### Rapid ART Barrier Ranking

Most of the frequently identified barriers impacting rapid ART initiation were considered highly important and highly feasible to change (eg, knowledge around the benefits of rapid ART initiation and skills-based training including the process of rapid HIV screening and confirmatory testing and motivational interviewing to foster rapid ART initiation [16]). The cost of the intervention with respect to the need to balance other clinical programs and internal benchmarks was determined to be difficult to change but also of low importance. Some characteristics of the Inner Setting (eg, HIV and LGBTQ+ stigma, competing priorities, and burnout) and Outer Setting (eg, lack of tangible incentives) were considered highly important but difficult to change. Although setting goals and receiving feedback on goals was considered highly feasible, it was considered less important (Table 2).

**Table 2.** Combined Modified Conjoint Analysis Results for Rapid ART Barriers Among Implementing HIV Testing and Treatment Sites in Memphis/Shelby County, Tennessee, 2023^a^

	Low Feasibility	High Feasibility
High Importance	Lack of tangible incentives Stigma related to being a government agency, dedicated HIV clinic, faith-based institution Competing priorities (eg, primary care for HIV-negative people, COVID and Mpox epidemics) Staff burnout Lack of provision of HIV care at some testing sites	Need for centrally located and easily accessible rapid ART model information Need for local program branding Social determinants of health, trauma, and stigma needs often take priority to rapid ART initiation Lack of networking and communication within and across HIV care and service organizations Lack of resources (eg, flexible scheduling templates, rapid HIV confirmatory test kits, ART starter pack samples) Available HIV and rapid ART knowledge/skills training is not routinely accessed or tracked Need to identify each organization's internal champion, opinion leader, and implementation leader Lack of venue to share successes and challenges with rapid ART and refine model
Low Importance	Balancing new programs and current internal program benchmarks (eg, client cycle time)	Lack of identification of program goals and feedback toward goals

Abbreviation: ART, antiretroviral therapy.

^a^Barriers are listed in no rank order within each quadrant.

For 2 of the 3 HIV testing sites not currently providing HIV medical care, the implementation climate of the Inner Setting, specifically, the current lack of provision of HIV medical care, was considered highly important but difficult to change. For the 1 site providing both HIV testing and care, the lack of a protocol for accepting and dispensing ART pharmaceutical samples was considered highly important and feasible to change (Table 2).

### Rapid ART Implementation Strategy Selection

For the barriers ranked highly important and feasible to change, 1 to 3 implementation strategies per CFIR Construct/Domain were identified (Table 3), again to balance comprehensiveness with feasibility. For example, the challenge of limited access to centrally located rapid ART model information was tackled by creating a standard operating procedures (SOP) document, which is now available on the Memphis EHE website (https://endhiv901.org). The SOP includes information on the purpose of and rationale for the toolkit, patient eligibility and operations (eg, laboratory testing, referral process from HIV testing to treatment sites, ART regimen selection), program evaluation metrics, recommended implementing staff training, and recommended stigma reduction activities.

**Table 3.** Highly Important and Highly Feasible Barriers and Implementation Strategies Identified in Memphis/Shelby County, Tennessee, 2023

Highly Important and Feasible Barriers	Implementation Strategy Identified
Lack of centrally located and easily accessible rapid ART model information	Standard operating procedures (SOP) document describing program components was developed Permission was obtained to include rapid ART program information on jurisdiction's Ending the HIV Epidemic website (endhiv901.org)
Lack of local program branding	A consultant from a locally owned branding and consulting company was hired to assist with program logo and social media toolkit development
Social determinants of health (SDoH), trauma, and stigma needs often take priority to rapid ART initiation	SOP document was developed that included: Resources/referral options for assistance with SDoH needs Recommendations on trainings on unconscious bias, cultural humility, and trauma-informed care Recommendations for stigma reduction activities
Lack of networking and communication within and across HIV care and service organizations Lack of venue to share successes and challenges with rapid ART and refine model	Quarterly in-person meetings among implementing sites were conducted and a steering committee consisting of 2 members per site was developed Each participating site executed a memorandum of understanding for rapid ART program activities
Lack of resources (eg, flexible scheduling templates, rapid HIV test confirmatory test kits, ART starter pack samples)	Sites not utilizing pharmaceutical samples for rapid ART initiation were linked to local pharmaceutical representatives for assistance in developing local pharmacy sample protocols
Available rapid ART knowledge is not routinely accessed or tracked	List of recommended trainings related to rapid ART initiation provided in written SOP
Need to identify each organization's internal champion/opinion leader and implementation leader	Site champions and implementation leaders were identified and their contact information was included in written SOP to facilitate referral of clients from testing to treatment sites

Abbreviation: ART, antiretroviral therapy.

Guidance for selecting rapid ART regimens was based on the *US DHHS Guidelines for the Use of Antiretroviral Agents in Adults and Adolescents with HIV* [3]. For sites not already utilizing medication samples, the SOP included contact information for local pharmaceutical company representatives for assistance in developing local site medication sample protocols.

Program evaluation metrics were collaboratively established by academic, implementing, and community partners, drawing on benchmarks identified by UNAIDS [17], the US Ending the HIV Epidemic Plan [18], and the local Memphis Ending the HIV Epidemic Plan (*End HIV 901*) [12]. We also considered the ease of data acquisition for measuring progress and the feasibility of achieving results compared to baseline data (Table 4).

**Table 4.** Performance Measures for Rapid ART Initiation in Memphis, Shelby County, Tennessee, 2024

Performance Measure^a^	Goal
Proportion of positive rapid HIV screening tests confirmed by a second rapid HIV confirmatory test	90%
Median time from positive HIV screening test to confirmatory HIV testing	1 d
Proportion of patients newly diagnosed with HIV who complete an HIV provider visit within 3 d	90%
Median time from HIV diagnosis to linkage	3 d
Proportion of patients newly diagnosed with HIV who initiate ART within 3 d	90%
Median time from HIV diagnosis to ART initiation	3 d
Median time from HIV diagnosis to viral suppression (viral load <200)	120 d
Disparities in these performance measures based on age, race/ethnicity, gender, or insurance status	No differences in above performance measures based on sociodemographic characteristics

Abbreviation: ART, antiretroviral therapy.

^a^Performance measures can all be measured using public health surveillance data.

The SOP training section included the training subject, where to locate the training, who should complete the training, and the recommended training frequency. Recommendations for several locally developed stigma reduction activities were made in the written SOP including training in cultural humility, unconscious bias, and trauma-informed care for all implementing site staff. Web-based trainings that were asynchronous were chosen so that implementing site staff could complete trainings as their schedule allows without being offsite for extended periods.

A programmatic logo was developed to accompany the SOP and address the CFIR Intervention Design domain and Intervention Design Quality and Packaging CFIR construct. Logo ideas were solicited via a crowdsourcing campaign among community partners. Idea submissions were voted on among implementing and community partners and the submission with most votes was selected to move forward for professional development by a locally owned branding consultant. The logo has been utilized on the program website, a social media toolkit, t-shirts for implementing partners, and for tabling at community events.

## DISCUSSION

We utilized process mapping and modified conjoint analysis to identify and rank local barriers to rapid ART initiation and identify locally relevant rapid ART strategies in Memphis. Our community-engaged approach allowed for partners to develop important relationships that facilitated honest discussions about local rapid ART barriers, rank barriers based on their importance and feasibility to change, encourage group brainstorming of implementation strategies to address highly ranked barriers, and create a sustainable collaborative partnership focused on rapid ART initiation in Memphis moving forward.

Our team bundled these implementation strategies to create a locally tailored rapid ART toolkit. Although several rapid ART initiation toolkits already exist [8–10], these toolkits serve as general guidance and need local adaptation. While some components of existing toolkits may be highly relevant in 1 setting, they may be less relevant in another setting. Our implementing and community partners expressed the need to develop toolkit components that fit the cultural and clinical context of Memphis and that are perceived as locally developed to facilitate buy-in and enable implementation.

For example, existing toolkits provide general guidance around the need for training in cultural humility, unconscious bias, and trauma-informed care without providing specific training details. To ensure that our rapid ART toolkit was perceived as internally developed, we sought to identify local training opportunities via the Tennessee Department of Health and Southeast AIDS Education and Training Center, which is housed at Vanderbilt University Medical Center in Nashville, Tennessee. The Southeast AIDS Education and Training Center is 1 of 8 regional Education and Training Centers across the country and provides educational opportunities aimed at increasing the size and strength of the HIV clinical workforce and meeting US Ending the HIV Epidemic goals [19].

Similarly, existing toolkits recognize stigma as an important barrier to rapid ART initiation but do not provide specific stigma reduction activity guidance. Although stigma was considered less feasible to change compared to other barriers, it emerged as one of the most frequently identified and important barriers in our work. Previous studies highlight the burden of stigma in the US South and the complex relationships between faith, HIV, and sexual/gender identity [20, 21]. For these reasons, we included guidance toward local stigma reduction activities in our toolkit. For example, failure to use person-first language can contribute to HIV stigma, resulting in suboptimal clinical outcomes such as unsuppressed viremia and poor mental health outcomes [22]. The Tennessee Department of Health and Tennessee Department of Mental Health and Substance Abuse Services have developed a language guidance document, which provide examples of non-stigmatizing language developed using the principles of person-first language, treating others the way they wish to be treated, and cultural humility [23]. This document was included in the section of the SOP with recommended rapid ART training subjects.

The Memphis *Snap Out Stigma* [24–26] project employed Photovoice to allow PWH to communicate their lived experiences with stigma through photography. Our implementing sites were invited to choose from a selection of Photovoice storyboards that were professionally printed by the study team and displayed in clinic areas and used during internal staff trainings around HIV stigma. This approach has been shown to encourage discussions about internalized stigma between PWH and their healthcare providers and support networks in Memphis, TN.

Although goal was considered less important in the modified conjoint analysis; the identification of rapid ART program goals was considered highly feasible for sites to measure their progress over time. The US Health Resources and Services Administration (HRSA) Ryan White HIV/AIDS Program has identified a performance measure portfolio aligned with the HIV care continuum [27]. However, linkage to HIV care and ART initiation is not currently included as an HRSA HIV performance measure. Although these performance measures are not definitively linked to Ryan White funding, they do provide measures that Ryan White–funded HIV care sites are required to report on and for which HRSA-funded sites are audited. The addition of time to ART initiation as an HRSA HIV performance measure could be a powerful external policy providing incentivization to organizations to prioritize rapid ART initiation.

Future work should address rapid ART barriers considered highly important but less feasible to change. One includes addressing the provision of rapid ART at implementing sites currently providing only HIV testing and not HIV treatment services. Task-shifting and task-sharing are 2 effective and cost-efficient models for delivering high-quality healthcare [28–31]. Task-shifting involves reallocating responsibilities from higher or more specialized levels of care to lower or less specialized levels. For example, the provision of ART starter packs can be delegated to other staff members, enabling physicians and advance practice providers to focus on tasks that require their specific expertise and licensure. Any clinician licensed to conduct a clinical assessment for rapid ART eligibility (assessment for intracranial opportunistic infections and cytomegalovirus retinitis) can dispense ART sample starter packs, including registered nurses and pharmacists. Task-sharing refers to cross-training staff serving in a single division or department to perform tasks conducted in another division or department. For example, health department staff hired in Maternal and Child Health could be cross-trained to perform tasks such as rapid ART initiation typically conducted by staff hired in the Sexual Health Program.

The second barrier noted to be highly important but less feasible to change that should be addressed in future work is staff burnout. HIV care providers are repeatedly exposed to patients experiencing significant trauma that, in turn, leads to vicarious trauma, compassion fatigue, and burnout. Trauma-informed care is an organizational approach to care delivery in which staff realize that trauma is prevalent, recognize how trauma impacts both patients and implementing site staff, and respond to trauma using this knowledge [32]. Existing toolkits address the role of trauma and trauma-informed care for PWH, but they do not address the impact of trauma and trauma-informed care interventions for staff at HIV testing and care organizations.

Although the implementation science methods used here aim to produce contextually relevant results across diverse settings, they also have limitations. Specifically, if the academic, implementation, and community partners do not include the voices of all critical scientific disciplines, local HIV service providers, and PWH, the barrier ranking activities and identification of important, feasible strategies may be biased.

## CONCLUSIONS

We identified strategies to overcome barriers to rapid ART initiation considered by implementing and community partners to be highly feasible and important in Memphis, Shelby County, Tennessee—a phase I Ending the HIV Epidemic priority jurisdiction located in the US South. These strategies were compiled into a toolkit that will be refined, evaluated, and disseminated in the second phase of this project. Importantly, we will continue to identify strategies aimed at long-term maintenance and sustainability, anticipating improvements in HIV linkage outcomes in Memphis that align with US Ending the HIV Epidemic goals. Similar IS strategies could be employed in other diverse settings to implement a local, contextually appropriate rapid ART initiation program.
